# IL‐1β and IL‐6 synergistically drive murine TFH cell differentiation and maintenance *in vitro*

**DOI:** 10.1111/imcb.70104

**Published:** 2026-03-31

**Authors:** Juliana Restrepo Munera, S Rameeza Allie

**Affiliations:** ^1^ Department of Cell and Biological Systems Penn State College of Medicine Hershey PA USA

**Keywords:** Cytokines, Differentiation, IL‐1β, IL‐6, *In vitro*, TFH cell

## Abstract

T follicular helper (TFH) cells are critical in supporting B‐cell responses, which ultimately establish long‐term protective immunity against infection and some cancers by producing protective B cells, including antibody‐secreting cells and memory cells. However, this same GC output can also promote autoimmune pathology. Therefore, TFH cells are at the core of humoral immunity, underscoring TFH cells as targets for long‐term protective and/or pathologic immune responses. Here we define conditions that promote physiologically relevant TFH cell differentiation *in vitro*. We demonstrate that in addition to T‐cell receptor (TCR) signaling, IL‐6 and IL‐1β individually and jointly promote TFH cell differentiation. These findings highlight key requirements for generating functional TFH cells, providing a model to study therapeutic impacts on TFH cells across diverse disease settings.

## Graphical abstract

We define a physiologically relevant *in vitro* system that demonstrates the combined function of T‐cell receptor signaling, IL‐6 and IL‐1β individually and jointly to promote TFH cell differentiation. These findings identify the essential requirements for generating functional TFH cells and clarify how cytokines shape their differentiation within the germinal center.

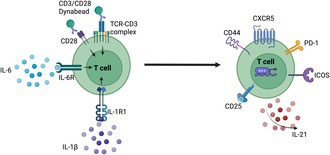

## INTRODUCTION

T follicular helper (TFH) cells are crucial to the adaptive humoral response, providing essential help to B cells. TFH cells are required for both formation and maintenance of the germinal center (GC), in which B cells undergo rounds of proliferation, maturation, selection, and differentiation.^1^ Interactions with TFH cells are critical for B cells to undergo these processes and their differentiation into memory B cells and antibody‐secreting cells (ASCs), both essential for long‐term immunity.^2^

TFH cell differentiation is a multi‐step process, requiring sequential cues from various antigen presenting cells (APCs) within secondary lymphoid organs. Initially, dendritic cells, an APC, present antigen to naïve T cells, initiating their commitment to the pre‐TFH lineage. At this stage, the emerging pre‐TFH cells acquire intermediate expression of B‐cell lymphoma 6 (Bcl‐6), chemokine receptor 5 (CXCR5), programmed cell death protein 1 (PD‐1) and inducible T‐cell co‐stimulator (ICOS).^3^ T cells then migrate to the T–B border where they will be presented with antigen by B cells, also APCs. These interactions enable pre‐TFH cells to complete their differentiation into mature TFH cells and subsequently enter the B‐cell follicle.^4^, ^5^ The differentiation of T cells into TFH cells requires the expression of the lineage‐defining transcription factor Bcl‐6,^6^ which is induced by T‐cell receptor (TCR) signaling^7^ and CD28 co‐stimulation.^8^ During this process, TFH cells must also upregulate CXCR5, which binds CXCL13, produced by stromal cells within the B cell follicle. Therefore, CXCR5 expression is essential for TFH cell function as it enables the directed migration of the TFH cells into the B‐cell follicle.^9^ Initial upregulation of CXCR5 and migration toward the B‐cell follicle is dictated by CD28 signaling. Once TFH cells enter the B‐cell follicle and/or the GC, ICOS becomes essential for their maintenance. Although, ICOS signaling is not required for initial TFH differentiation, it is critical for sustaining the TFH program and consequently for maintaining GCs.^8^ Multiple co‐stimulatory signals reinforce the TFH identity and support their continued localization within the follicle, where they aid in formation and maintenance of the GC, which results in the selection of B cells expressing high‐affinity B‐cell receptors.

Although TFH cells provide essential signals and cytokines to help B cells, they themselves require reciprocal help from B cells, as well. B cells promote TFH cells by (1) presenting antigen for TCR signaling,^10^ (2) providing stimulatory ligands that activate CD28 and ICOS^11^, ^12^ and (3) by providing cytokines which promote TFH cell differentiation and function. Interleukin (IL)‐6 signaling has previously been shown to be essential for the induction of Bcl‐6 and CXCR5.^13^ B cells can serve as a key source of IL‐6 within the GC microenvironment, supporting TFH cell function.^14^ More recently, B cells have been identified as producers of IL‐1β within the GC,^15^ underscoring a potential requirement for IL‐1 signaling for TFH differentiation and function, as well. Both dendritic cells in the T‐cell zone^16^, ^17^, ^18^ and B cells within the GC^15^ appear well positioned to provide IL‐1β, thereby supporting the development and maintenance of the TFH program.

IL‐1 signaling broadly supports T‐cell activation and contributes to the differentiation of multiple T‐cell subsets.^19^, ^20^ Particularly, TFH cell function has been shown to depend on signaling through the agonistic receptor for IL‐1, IL‐1R1, which can be engaged by both IL‐1β and IL‐1α. *In vitro* stimulation of isolated TFH cells with IL‐1β promotes TFH cell function, evidenced by increased IL‐21 production, whereas pharmacological blockade of IL‐1 signaling inhibited TFH cell function.^21^ *In vivo*, T‐cell specific depletion of IL‐1R1 is also observed to result in decreased TFH cell and GC B cell numbers after influenza infection.^15^ Similarly, after OVA‐immunization, mice lacking IL‐1R1 in T cells exhibit decreased frequencies of TFH and GC B cells.^22^

Here we demonstrate that naïve T cells from wild‐type C57BL/6J mice (WT mice) can be differentiated *in vitro* through coordinated stimulation of T‐cell coreceptors and co‐stimulatory receptors, along with cytokine receptors. Currently, *in vitro* differentiatio*n* of TFH cells relies on co‐culturing cells from mice that have antigen‐specific TCRs with APCs that have cognate MHC‐II complexes.^23^, ^24^ While the individual contributions of IL‐6 and IL‐1β in promoting TFH cell development and function have been previously described, this study investigates how IL‐6 and IL‐1β, individually and jointly, shape TFH differentiation. We dissect multiple stages of TFH development, including T‐cell activation, transcriptional programming, acquisition of effector functions and maintenance, and how each signal contributes to these steps. Ultimately, we show that IL‐6 and IL‐1β collaborate to optimize TFH development, enhancing their functional capacity and the acquisition of migratory features, which may induce their localization within the GC microenvironment *in vivo*.^9^, ^15^ In addition to defining mechanistic requirements for TFH cell development, this work established an experimental framework for evaluating TFH differentiation *in vitro*.

## RESULTS

### *In vitro* T‐cell activation enhanced with IL‐6 and IL‐1β signaling

Upon activation by antigen exposure in influenza infected mice, CD4^+^ T cells upregulate the agonistic receptor for IL‐1 signaling, IL‐1R1. In the mediastinal lymph node of influenza infected mice, the majority of the activated cells, demarcated by high CD44 expression, are TFH cells.^15^ In addition to CD44, CD25 is also upregulated early during T‐cell activation and is commonly used as an activation marker.^25^ *In vivo*, however, TFH cells downregulate CD25 as they enter the GC, because IL‐2 signaling antagonizes Bcl‐6. In contrast, *in vitro* TFH differentiation does not involve migration into distinct anatomical areas; therefore, generation of TFH‐like cells in culture does not exhibit CD25 downregulation, allowing the use of CD25 expression as a general activation marker in these cultures.^26^ To model TFH differentiation, we isolated splenic CD4^+^ T cells from naïve WT mice and stimulated them with defined combinations of signals designed to drive differentiation of naïve CD4^+^ T cells to TFH cells *in vitro*. Each stimulation cocktail (Table 1) contained TCR stimulation combined with essential cues including cytokines that promote the TFH lineage commitment.

**Table 1:** Stimulation groups for *in vitro* differentiation of naïve T cells into TFH cells.

Media Change Day	Stimulation		
	D3	D6	D9
Group 1 (G1)	Media only		
Group 2 (G2)	CD3/CD28 beads		
Group 3 (G3)	CD3/CD28 beads IL‐6 ICOSL		
Group 4 (G4)	CD3/CD28 beads IL‐6 IL‐1ß		
Group 5 (G5)	CD3/CD28 beads IL‐1ß ICOSL		
Group 6 (G6)	CD3/CD28 beads IL‐6 IL‐1ß ICOSL		
Group 7 (G7)	CD3/CD28 beads IL‐6		
Group 8 (G8)	CD3/CD28 beads ICOSL		
Group 9 (G9)	CD3/CD28 beads IL‐1ß		

*Note:* Isolated naïve T cells were incubated for a total of either 6 or 9 days. Stimulation with treated culture media was started at day 0 (D0) and changed on D3 before staining on D6, or changes at D3 and D6 for staining at D9. T cells were stimulated with CD3/28 dynabeads (1:1 beads to cell ratio), rIL‐6 (100 ng/mL), rICOSL (200 ng/mL) and/or IL‐1β (20 ng/mL).

Before assessing the differentiation of naïve T cells into TFH cells, we first evaluated how the different stimulation conditions influenced early T‐cell activation. We used live, singlet, CD4^+^ lymphocytes and quantified activation based on the frequencies of CD25^+^ CD44^+^ cells (Supplementary figure 1a). As expected, culturing T cells in media alone (group 1) (Table 1) failed to induce activation (Supplementary figure 1b, c), which resulted in poor survival of lymphocytes in group 1, both in the 6‐ and 9‐day cultures (Supplementary figure 1d, e). As group 1 lacks sufficient survival and does not provide a meaningful biological comparison, we excluded it from subsequent statistical analyses and instead used group 2 (Table 1), as a statistical control.

**Figure 1:**
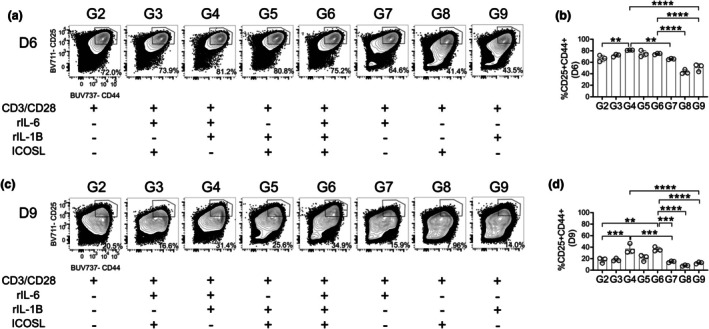
*In vitro* activation of CD4^+^ T cells. Cultured isolated splenic CD4^+^ T cells were gated on live, singlet lymphocytes (Supplementary figure 1). CD25^+^ CD44^+^ T cells were gated from live cells after culturing with varying stimuli (Table 1) for **(a)** 6 days or **(c)** 9 days. CD25^+^ CD44^+^ T‐cell numbers were quantified from **(b)** 6 day and **(d)** 9 day cultures. Data are representative of three biological experiments each with three technical replicates **(a–d)**. Graphs show individual points and mean ± SD of technical replicates from a representative experiment **(b, c)**. Selected statistics are displayed **(b, c)** and all comparisons can be found on Table 3. *P < 0.05, **P < 0.01, ***P < 0.001, and ****P < 0.0001.

We first assessed whether TCR stimulation alone was sufficient to induce T‐cell activation in culture. The CD3/CD28 Dynabead stimulation (group 2) reliably generated a CD25^+^ CD44^+^ activated population, during a 6‐day culture. Across all groups, additional stimuli (groups 3–9) (Table 1), did not further increase the magnitude of early activation, in the 6‐day culture (Figure 1a, b). However, by Day 9, we see significant reductions in the frequency of activated T cells across most conditions. At Day 9, TCR stimulation alone (group 2) was insufficient to sustain activation and the addition of IL‐6 (group 7), ICOSL (group 8) or IL‐1β (group 9) (Table 1) individually also failed to maintain activated T cells (Figure 1c, d). Similarly, the combination of TCR stimulation with IL‐6 and ICOSL (group 3) or ICOSL and IL‐1β (group 5) (Table 1) did not support prolonged T‐cell activation (Figure 1c, d). In contrast, T‐cell activation is observed only when TCR stimulation is provided with IL‐6 and IL‐1β (group 4) or with IL‐6, IL‐1β and ICOSL (group 6) (Figure 1c, d, Table 1). As the difference in groups 4 and 6 is the addition of ICOSL in group 6, and no significant difference in activation was observed between these two groups, we conclude that ICOS signaling is not further enhancing the maintenance of activated T cells under these conditions. Therefore, TCR stimulation is sufficient to induce T‐cell activation and IL‐6 and IL‐1β are cooperating to support prolonged activation, each enhancing the other's effect as seen in groups 4 and 6.

### TFH development determined by upregulation of transcriptional regulator Bcl‐6

**Figure 2:**
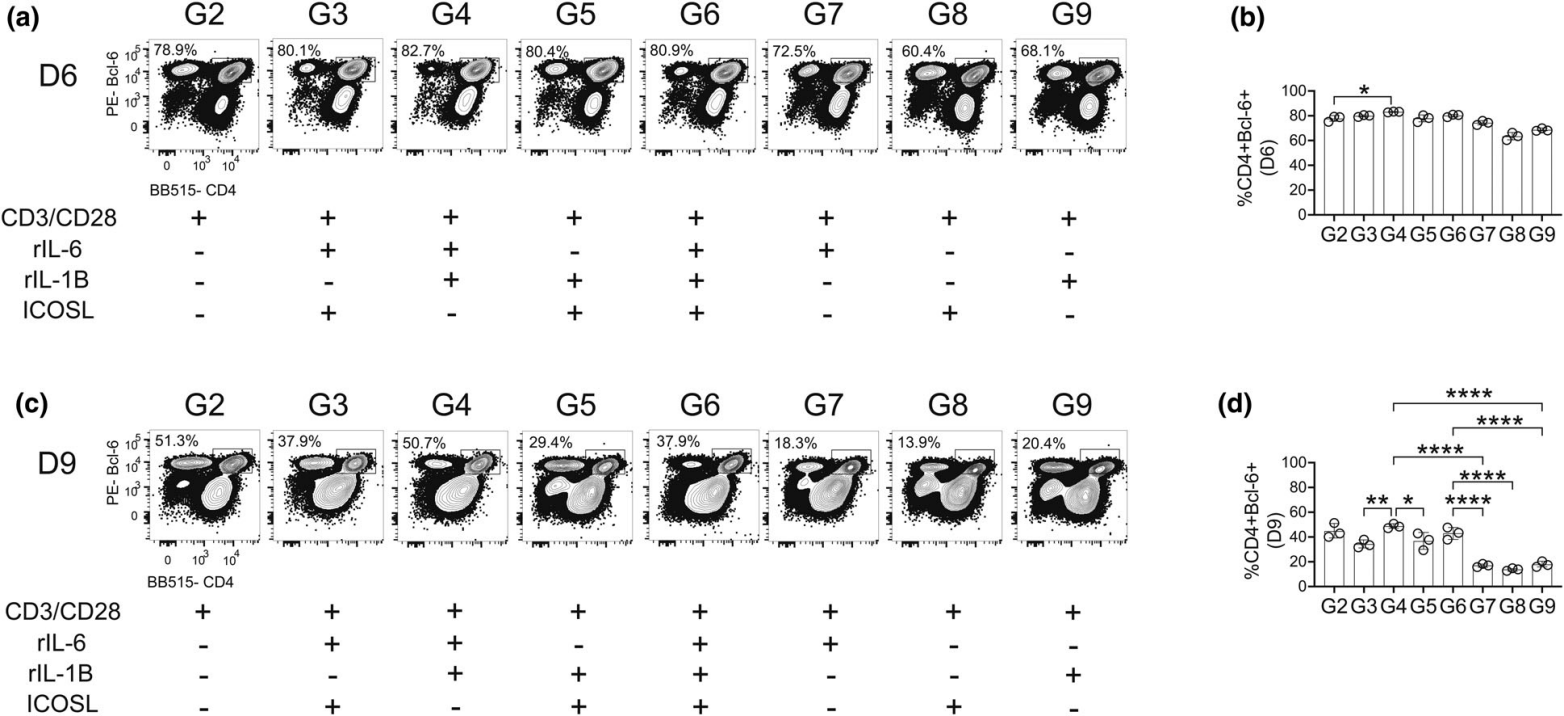
Bcl‐6 transcription factor upregulation in stimulated naïve T cells. Cultured isolated splenic CD4^+^ T cells were gated on live, singlet lymphocytes (Supplementary figure 1). CD4^+^ Bcl‐6^+^ T cells were **(a)** gated from live cells and **(b)** quantified as frequency of live cells following 6 days of culture in nine different variations of stimuli. CD4^+^ Bcl‐6^+^ T cells were **(c)** gated from live cells and **(d)** quantified as frequency of live cells following 9 days of culture in nine different variations of stimuli. Data are representative of three biological experiments each with three technical replicates **(a–d)**. Graphs show individual points and mean ± SD of technical replicates from a representative experiment **(b, c)**. Selected statistics are displayed **(b, c)** and all comparisons can be found on Table 3. *P < 0.05, **P < 0.01, ***P < 0.001, and ****P < 0.0001.

To further assess the capacity of naïve T cells to differentiate into TFH cells, we examined Bcl‐6 expression in CD4 T cells. TCR stimulation alone (group 2) is sufficient to induce significant expression of Bcl‐6 in the 6‐ and 9‐day culture (Figure 2), consistent with prior studies showing that strong TCR avidity can drive T cells toward the TFH lineage.^27^, ^28^ The use of CD3/CD28 beads bypasses the need to activate the TCR through binding to an MHC‐II complex, by stimulating the TCR through CD3 and also adding a co‐stimulatory signal through CD28.^29^ Therefore, cells in groups 2–9 (Table 1), all of which are being stimulated using the CD3/CD28 beads, are experiencing strong TCR stimulation, explaining the uniformly high Bcl‐6 expression across all groups, primarily in the 6‐day culture (Figure 2a, b). After 9 days in culture, groups 2, 4, and 6 (Table 1) maintained the highest frequency of Bcl‐6^+^ CD4 T cells (Figure 2c, d). Although all groups upregulate Bcl‐6 expression, these three groups showed the most sustained and abundant expression at both timepoints (Figure 2b, d). However, as Bcl‐6 expression alone is insufficient to define bona fide TFH cells, additional scrutiny of phenotype and function is required to confirm TFH differentiation. Therefore, while TCR stimulation is sufficient to induce Bcl‐6 expression, which is not further upregulated by individual or combined IL‐6, IL‐1β or ICOS signaling, further analysis is necessary to determine the optimal combination of signals required in generating fully functional TFH cells.

### IL‐6 and IL‐1β collaborate in differentiation of naïve T cells into TFH cells

**Figure 3:**
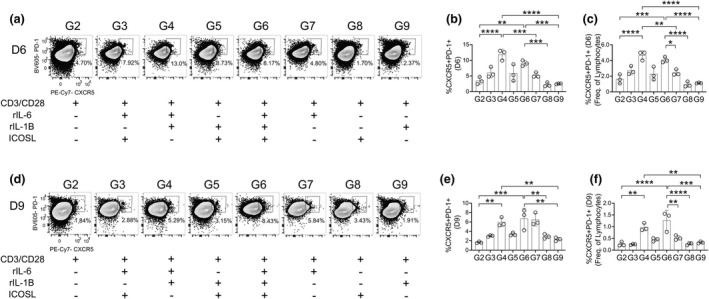
Differentiating naïve T cells into TFH cells. **(a)** CXCR5^+^ PD‐1^+^ T cells (TFH) were gated from CD4^+^ Bcl‐6^+^ T cells (Figure 2) after 6 days of stimulation. TFH cells from 6‐day culture were quantified as frequency of **(b)** CD4^+^ Bcl‐6^+^ T cell and as **(c)** frequency of live lymphocytes. **(d)** CXCR5^+^ PD‐1^+^ T cells (TFH) were gated from CD4^+^ Bcl‐6^+^ T cells (Figure 2) after 9 days of stimulation. TFH cells from 9‐day culture were quantified as frequency of **(e)** CD4^+^ Bcl‐6^+^ T cell and as **(f)** frequency of live lymphocytes. Data are representative of three biological experiments each with three technical replicates **(a–f)**. Graphs show individual points and mean ± SD of technical replicates from a representative experiment **(b, c, e, f)**. selected statistics are displayed **(b, c, e, f)** and all comparisons can be found on Table 3. *P < 0.05, **P < 0.01, ***P < 0.001, and ****P < 0.0001.

Bcl‐6 expression represents the early steps of TFH cell differentiation; however, additional surface proteins must be upregulated to further establish TFH cell differentiation and function, including CXCR5 and PD‐1.^30^, ^31^ Therefore, we examined for CXCR5^+^ PD‐1^+^ co‐expression within the Bcl‐6^+^ CD4^+^ T‐cell population (Figure 3). To accurately gate this population, we examined CXCR5^+^ PD‐1^+^ co‐expression across all groups, including group 1 (Supplementary figure 2a) which was treated with media only. Although CXCR5^+^ PD‐1^+^ cells do not constitute a substantial population in group 1, due to limited survival, a clearly defined CXCR5^+^ PD‐1^+^ double‐positive population is still detectable, allowing for the accurate placement of the gate. Although group 2, with TCR stimulation only, induced significant activation and Bcl‐6 expression, the frequency of CXCR5^+^ PD‐1^+^ cells remained low, indicating that TCR stimulation alone is insufficient to generate TFH cells in either 6‐day (Figure 3a–c) or 9‐day (Figure 3d–f) cultures. At 6 days, the highest frequencies of CXCR5^+^ PD‐1^+^ cells were observed in groups 4 and 6 (Figure 3b, Table 1). By 9 days, groups 4, 6 and 7 (Table 1), exhibited the greatest enrichment of CXCR5^+^ PD‐1^+^ TFH cells (Figure 3e). Together, these data indicate that T cells stimulated with IL‐6 combined with IL‐1β promote TFH cell differentiation at 6 days in culture, while prolonged stimulation in culture (9‐day) suggests that TCR stimulation with IL‐6 stimulation is sufficient to support TFH cells.

As the analyses in Figure 3b, e examined frequencies of CXCR5^+^ PD‐1^+^ cells among Bcl‐6^+^ CD4^+^ T cells, they do not account for differences in cell viability or total Bcl‐6 expression across groups. Therefore, to determine the overall TFH cell output per culture, we also quantified the frequency of CXCR5^+^ PD‐1^+^ among total lymphocytes. This approach allowed us to examine the frequency of singlet, live, CD4^+^ Bcl‐6^+^ CXCR5^+^ PD‐1^+^ cells within the lymphocytes and also allows us to determine the frequency of total live TFH cells per well (Supplementary figures 1a and 2b). We observe modest increases in TFH cells when groups 3, 5 and 7 are compared with the control group 2 (Table 1), in the 6‐day culture (Figure 3c), demonstrating that TCR stimulation combined with IL‐6 and ICOSL (group 3), IL‐1β and ICOSL (group 5) and IL‐6 alone (group 7) can promote low‐level TFH differentiation. However, the most significant increases in TFH cells were observed in groups receiving TCR stimulation with IL‐6 and IL‐1β (group 4) and IL‐6, IL‐1β and ICOSL (group 6) (Figure 3c, Table 1). Additionally, while IL‐6 alone (group 7) increased the frequency of CXCR5^+^ PD‐1^+^ co‐expression among the Bcl‐6^+^ CD4^+^ T cells at 9 days (Figure 3e), this was not reflected when examining total TFH cells (Figure 3f). Instead, at 9 days post stimulation, IL‐6 combined with IL‐1β (groups 4 and 6) (Table 1) remained the strongest promoter of TFH cell differentiation, when examining total TFH cells (Figure 3f).

### IL‐6 and IL‐1β in early programming vs. maintenance of TFH cells

**Figure 4:**
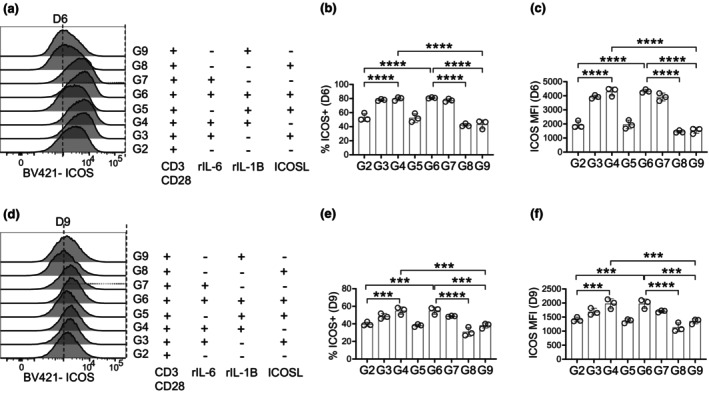
ICOS expression is dependent on IL‐6 signaling. CD4^+^ Bcl‐6^+^ T cells (Figure 2) were **(a)** gated for ICOS expression and quantified as **(b)** frequency of CD4^+^ Bcl‐6^+^ and **(c)** on a per cell basis after 6 days of stimulation. **(d)** ICOS expression and quantified as **(e)** frequency of CD4^+^ Bcl‐6^+^ and **(f)** on a per cell basis after 6 days stimulation. Data are representative of three biological experiments each with three technical replicates **(a–f)**. Graphs show individual points and mean ± SD of technical replicates from a representative experiment **(b, c, e, f)**. selected statistics are displayed **(b, c, e, f)** and all comparisons can be found on Table 3. *P < 0.05, **P < 0.01, ***P < 0.001, and ****P < 0.0001.

To identify the signals required to generate TFH cells, *in vitro*, that can resemble GC‐TFH cells in their ability to persist, we examined ICOS expression on Bcl‐6^+^ CD4^+^ T cells. ICOS is essential early in TFH cell development for follicular entry, while PD‐1 is required later for localization to the GC.^32^ In both 6‐ and 9‐day cultures, IL‐6 induced the highest upregulation of ICOS, both by frequency of ICOS^+^ cells (Figure 4a, b, d, e) and expression level per cell (Figure 4c, f). This pattern is consistent across all groups containing IL‐6 (groups 3, 4, 6 and 7) (Table 1), after 6‐ and 9‐ days of stimulation. In contrast, groups lacking IL‐6 (groups 2, 5, 8 and 9) (Table 1) showed markedly lower ICOS expression. These data, along with our findings in Figure 3, demonstrate that IL‐6 is required for early TFH programming, especially for the upregulation of ICOS. In contrast, IL‐1β primarily contributes to T‐cell activation; however, the combined signaling of IL‐6 and IL‐1β is essential to sustain TFH cell maintenance. This cooperative effect highlights the importance of continuous cytokine support when culturing TFH cells for *in vitro* studies.

### *In vitro* TFH cell differentiation determined by function

**Figure 5:**
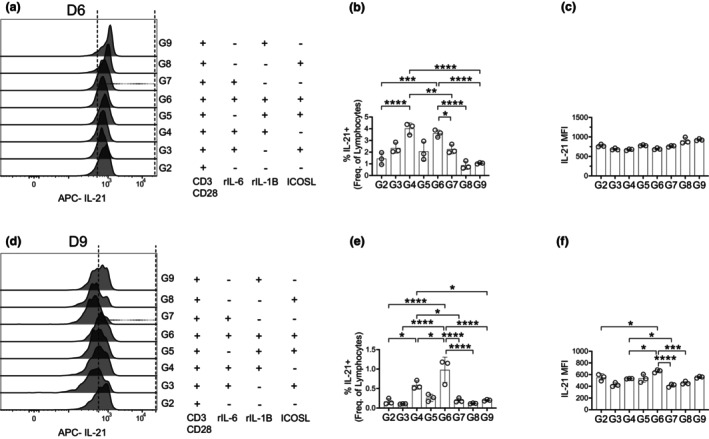
IL‐21 expression by TFH cells *in vitro*. CD4^+^ Bcl‐6^+^ T cells were gated from live singlet lymphocytes (Supplementary figure 1). TFH cells (Figure 3) produced from a 6‐day were gated for **(a)** IL‐21 and quantified as **(b)** frequency of live lymphocytes and **(c)** IL‐21 expression on a per cell basis. TFH produced from a 9‐day were gated for **(d)** IL‐21 and quantified as **(e)** frequency of live lymphocytes and **(f)** IL‐21 expression on a per cell basis. Data are representative of three biological experiments each with three technical replicates **(a–f)**. Graphs show individual points and mean ± SD of technical replicates from a representative experiment **(b, c** and **e, f)**. Selected statistics are displayed (**b, c** and **e, f**) and all comparisons can be found on Table 3. *P < 0.05, **P < 0.01, ***P < 0.001, and ****P < 0.0001.

TFH cells are essential for the GC to drive B‐cell proliferation, affinity maturation and differentiation into memory cells and ASCs. These functions require more than just the presence of TFH cells, but also their ability to deliver helper cytokines and signals. To further assess the functional capacity of *in vitro*‐generated TFH cells, we examined their production of IL‐21. To promote the intracellular enrichment of IL‐21, we incubated the T cells with Brefeldin A to inhibit the release of IL‐21, allowing for intracellular staining. A fluorescence‐minus one (FMO) control was used to determine the placement of the positive IL‐21 gate (Supplementary figure 2c) and IL‐21^+^ cells were gated from Bcl‐6^+^ CD4^+^ CXCR5^+^ PD‐1^+^ TFH cells (Supplementary figure 1a). IL‐21 production by TFH cells was comparable across groups (Figure 5a). However, assessing IL‐21^+^ TFH cells as a proportion of total lymphocytes allowed evaluation of how each stimulation condition affected the total yield of IL‐21‐producing cells. After a 6‐day culture, groups 4 and 6 showed the highest frequencies of IL‐21 producing TFH cells, with modest increases in groups 3, 5 and 7 (Figure 5b, Table 1). The mean fluorescence intensity (MFI) confirmed that on a per cell basis, TFH cells across groups produced IL‐21 at a similar level, while there are differences in frequency of total IL‐21^+^ TFH cells (Figure 5a, c). Similarly, the frequency of IL‐21^+^ cells were similar across all groups in a 9‐day culture (Figure 5d), however, when assessed as frequency of total lymphocytes, group 6 (TCR, IL‐6, IL‐1β and ICOS) exhibited the highest frequency of IL‐21^+^ TFH cells, surpassing even group 4 (Figure 5e), while also expressing higher IL‐21 on a per cell basis (Figure 5f).

Across our analyses of total TFH cells and IL‐21 producing TFH cells, we observe that after 6 days of stimulation, TCR, IL‐6 and IL‐1β (group 4) was the most effective in generating TFH cells *in vitro*, from naïve T cells (Figures 3c and 5b). However, by 9 days, the addition of ICOSL signaling (group 6) further enhanced TFH differentiation beyond the levels in group 4 (Figures 3f and 5e). Thus, while IL‐6 and IL‐1β drive the early formation of TFH cells, these data further supports the role of ICOS in sustaining the functional TFH population over time. Overall, our findings indicate that IL‐6 and IL‐1β cooperate to induce early TFH cell differentiation and function. Collectively, these results suggest a sustained requirement for these cytokine signals throughout all stages of T‐cell activation and differentiation into TFH cells and their associated function, and we hypothesize that these signals are also required *in vivo*.

## DISCUSSION

Helper T (Th) cells, as their name suggests, provide crucial aid to other cells whose roles lie in providing protection. TFH cells, a specialized subset of Th cells that assist GC B cells, are indispensable in the generation of memory B cells and ASCs, both of which mediate long‐term humoral immunity. Given the importance of these cells in protective responses, we aimed to establish a model in which TFH cells can be generated *in vitro*, thereby providing a platform for their cellular and molecular analysis. Here, we present a model in which TFH cells are generated *in vitro*, through combined TCR and cytokine signaling. Further, we highlight the cytokine signals required for both TFH cell formation and maintenance. These cytokine signals, including IL‐6 and IL‐1β, have been previously described in early differentiation of TFH cell fate through signaling in the T‐cell zone. More recent work has also identified these cytokines within the GC and demonstrated their roles in promoting GC formation. As our findings reveal the role of IL‐6 and IL‐1β in TFH cell maintenance, *in vitro*, this raises the question of how these signaling mechanisms translate to TFH maintenance *in vivo*.

We find that IL‐1β supports cell viability throughout all 9 days of culture (Supplementary figure 1d, e), whereas IL‐6 is less effective at maintaining cell survival. These findings are consistent with *in vivo* prior studies showing that IL‐1β specifically enhances antigen‐specific T‐cell survival and proliferative capacity.^33^ In contrast, IL‐6 promotes the expression of ICOS, a requirement for TFH cell maintenance, which IL‐1β alone does not (Figure 4a–c). Overall, IL‐6 signaling in group 7 is sufficient to induce T‐cell activation (Figure 1b), Bcl‐6 expression (Figure 2b), and upregulation of functional markers of TFH cells (Figures 3c and 4b, c), however, IL‐6 signaling alone is insufficient to maintain TFH cells over prolonged stimulation. Although not to the same extent as IL‐6 alone (group 7), IL‐1β stimulation alone (group 9) is also able to induce T‐cell activation and promote early Bcl‐6 expression, but it is unable to upregulate functional markers of TFH cells during early differentiation. Together, these findings suggest that IL‐1β primarily supports T‐cell activation and proliferation, while IL‐6 instructs early T‐cell fate toward a TFH phenotype. Thus, IL‐6 and IL‐1β have distinct, nonredundant roles in early TFH development. However, neither cytokine alone is sufficient to generate fully functional TFH cells. Instead, their combined signaling is required for TFH differentiation, maintenance and function. This observed requirement *in vitro* strongly implies that both IL‐6 and IL‐1β must be present within the GC microenvironment, which is supported by previous studies identifying GC B cells as a major source of IL‐6 (Arkatkar *et al*.) and IL‐1β (Restrepo Munera *et al*.). These findings further emphasize the noncanonical role of B cells as helper cells within the GC.

The notable importance of these findings lies in the establishment of an *in vitro* model that produces TFH cells closely resembling GC‐TFH cells. This system enables interrogation of TFH biology, including signaling pathways, metabolic programming and cell cycle regulation, across distinct developmental stages. Importantly, the model uniquely distinguishes between early TFH cells and GC‐TFH cells: 6‐day culture yields cells representing pre‐TFH cells poised at the T‐B border outside the follicle, whereas a longer 9‐day culture produces fully established TFH cells that are representative of GC‐TFH cells. This conclusion is highlighted through ICOS expression and its function in driving TFH cells at later time points. ICOS signaling is essential for TFH cell maintenance rather than initial TFH differentiation.^8^ As ICOS expression is increased significantly by Day 6 in culture (Figure 4a–c), this would explain why during the early formation of TFH cells, by 6‐ days of culture, stimulation with TCR, IL‐6, IL‐1β and ICOS (group 6) is comparable to stimulations with TCR, IL‐6 and IL‐1β (group 4) when examining TFH frequency (Figure 3c) and TFH cell function (Figure 5b). The increase in ICOS expression by Day 6, followed by increased TFH cell frequency (Figure 3f) and IL‐21^+^ TFH cells (Figure 5e) in group 6 compared with group 4 in the 9‐day culture, suggests that ICOS signaling is promoting TFH cells at this later time point, similar to how ICOS promotes GC‐TFH cells. This resolution positions this model as a transformative tool for advancing mechanistic studies on TFH function.

This physiologically relevant TFH provides a controlled system in which we can explore the roles of TFH cells in various disease settings, including cancer, autoimmunity and infection. Peripheral TFH‐like cells have been associated with improved outcomes in several cancers, likely due to their support of ASC formation in tertiary lymphoid structures (TLS).^34^ Conversely, TFH cells are implicated for their pathogenic roles in autoimmune diseases. In systemic lupus erythematosus (SLE), TFH cells drive the generation of autoreactive B cells within GCs and promote formation of TLS in peripheral tissues, leading to sustained local antibody production.^35^ Given the broad influence of TFH cells on B‐cell responses, the understanding of the mechanisms governing their differentiation and function, in the context of these diseases, is critical to identifying therapeutic strategies to either enhance or suppress TFH function.

We acknowledge that this reductionist approach does not fully capture the complex interactions of other factors and cell types within the B‐cell follicle. However, the cytokines used in this study were deliberately selected based on published research that demonstrates their impact on various stages of TFH differentiation *in vivo*.^8^, ^13^, ^15^ As such, the study effectively highlights the key signals required for *in vitro* TFH cell differentiation, grounded in *in vivo* findings, which provides a platform with significant translational potential.

While the experiments presented were performed on murine cells, it would be of interest to determine if TFH cells can be generated *in vitro* from isolated human T cells. Such analysis would determine if the stimuli that aid in generating and maintaining TFH cells in mice, *in vitro*, are translated in humans. If the proposed model can generate human TFH cells. It would further validate its potential for high‐throughput studies, particularly in drug development.

The findings in this study present a robust methodology for differentiating naïve T cells into physiologically relevant TFH cells, *in vitro*. This system not only facilitates the acquisition of mechanistic insight into TFH cell biology but also provides valuable information on the signals they may receive *in vivo*. Furthermore, the model offers significant translational value by serving as a platform for testing pharmacological agents that modulate TFH viability, maintenance, and effector function.

## METHODS

### Mice

C57BL/6J mice were purchased from The Jackson Laboratory (Bar Harbor, ME). Mice were bred at the Pennsylvania State College of Medicine vivarium. Both female and male mice were used, with only one sex used for a single biological replicate. Spleens were harvested from mice between 6 and 8 weeks of age.

### Organ processing

Spleens were isolated from 4 to 5 mice of the same sex, and mechanically processed to generate pooled single‐cell suspension, followed by red blood cell lysis in Ack Lysis Buffer (150 mM NH4Cl, 10 mM KHCO3 and 0.1 mM EDTA).

### T‐cell isolation and *in vitro* culture

T cells were isolated from naïve C57BL/6J spleens utilizing immunomagnetic negative selection kit (STEMCELL Technologies, Cambridge, MA). Cells were seeded in round‐bottom 96‐well plates at 2 $\times 10^{5}$ cells/well and culture in complete plasma cell media (RPMI supplemented with 10% FBS, 0.5% Penicillin (100×), 0.5% Streptomycin (100×), 0.7% glucose, 1% HEPES pH 7.4 (1 M), 1% L–Glutamine (200 mM), 0.15% sodium bicarbonate, 1.2% nonessential amino acids (100×), 1.2% vitamins (100×), 1% sodium pyruvate (100 mM), 0.1% 2–mercaptoethanol (1000×, 55 mM)). Cells were stimulated with mouse CD3/28 Dynabeads, recombinant IL‐6 (rIL‐6), recombinant ICOSL (rICOSL), recombinant mouse IL‐1β (rIL‐1β) (Table 1). Cells were cultured for either 6 or 9 days at 37°C in 5% CO_2_, with media changes every 3 days.

### Cell staining and flow cytometry

Cells were incubated with Brefeldin A (BFA) (10 μg/mL) for 5 hours at 37°C prior to staining. Cells were stained with fluorochrome‐conjugated antibodies (Table 2) titrated in 2% staining was buffer (SWB; 2% fetal bovine serum in PBS). Cell fixation and permeabilization, for intracellular staining, was performed using the Foxp3/Transcription factor buffer set (eBiosciences). The BD FACSSymphony (BD Biosciences, Franklin Lakes, NJ) was used to perform flow cytometry.

**Table 2:** List of antibodies used for flow cytometry.

Antibodies and Recombiant Proteins					
	Marker	Fluorophore	Clone	Isotype	Catalog #
Flow Cytometry	CD4	AF488	GK1.5	Rat IgG2b, κ	100423
	CD25	BV711	PC61	Rat IgG1, λ	102049
	CD185 (CXCR5)	PE‐Cy7	L138D7	Rat IgG2b, κ	145516
	Bcl‐6	PE	IG191E/A8	Mouse / IgG1	648304
	CD279 (PD‐1)	BV605	RMP1‐30	Rat IgG2a, κ	135219
	CD44	BUV737	IM7	Rat IgG2b, κ	612799
	ZOMBIE NIR Dye				77184
	CD278 (ICOS)	BV421	C398.4A	Armenian Hamster IgG	313523
	IL‐21	APC	FFA21	Rat IgG2a, κ	17‐7211‐82

*Note*: All comparisons and respective *P*‐values reported, along with statistical test performed.

### Statistical analysis

GraphPad Prism software (Version 10, GraphPad Software, Inc.) was used to perform all data analysis. The data displayed in all graphs are representative of mean ± standard deviation (SD), with each point on the graph representing a technical replicate. All experiments were replicated at least three times, with each biological replicate containing three technical replicates. Ordinary one‐way analysis of variance (ANOVA) with Turkey's multiple comparison test was used for comparison between groups. Comparisons were considered to be statistically significant when *P*‐values were <0.05. All comparisons are provided for Figures 1, 2, 3, 4, 5 (Table 3) and Supplementary figure 1 (Table 4).

**Table 3:** Statistics for main Figures 1, 2, 3, 4, 5.

Figure	Subfigure	*P*‐value		Stats Test
1	B	G2 vs G3 *P* = 0.7138 G2 vs G4 *P* = 0.0074 G2 vs G5 *P* = 0.2379 G2 vs G6 *P* = 0.2740 G2 vs G7 *P* > 0.9999 G2 vs G8 *P* < 0.0001 G2 vs G9 *P* = 0.0032 G3 vs G4 *P* = 0.1623 G3 vs G5 *P* = 0.9806 G3 vs G6 *P* = 0.9895 G3 vs G7 *P* = 0.5436 G3 vs G8 *P* < 0.0001 G3 vs G9 *P* = 0.0001	G4 vs G5 *P* = 0.5741 G4 vs G6 *P* = 0.5194 G4 vs G7 *P* = 0.0042 G4 vs G8 *P* < 0.0001 G4 vs G9 *P* < 0.0001 G5 vs G6 p > 0.9999 G5 vs G7 p = 0.1489 G5 vs G8 *P* < 0.0001 G5 vs G9 *P* < 0.0001 G6 vs G7 p = 0.1738 G6 vs G8 *P* < 0.0001 G6 vs G9 *P* < 0.0001 G7 vs G8 *P* < 0.0001 G7 vs G9 p = 0.0057 G8 vs G9 p = 0.2957	Ordinary one‐way ANOVA, Turkey's multiple comparison test
	D	G2 vs G3 *P* > 0.9999 G2 vs G4 *P* = 0.0008 G2 vs G5 *P* = 0.9351 G2 vs G6 *P* = 0.0011 G2 vs G7 *P* = 0.9927 G2 vs G8 *P* = 0.1903 G2 vs G9 *P* = 0.8660 G3 vs G4 *P* = 0.0010 G3 vs G5 *P* = 0.9668 G3 vs G6 *P* = 0.0014 G3 vs G7 *P* = 0.9806 G3 vs G8 *P* = 0.1500 G3 vs G9 *P* = 0.7998	G4 vs G5 p = 0.0071 G4 vs G6 p > 0.9999 G4 vs G7 p = 0.0002 G4 vs G8 *P* < 0.0001 G4 vs G9 *P* < 0.0001 G5 vs G6 *P* = 0.0100 G5 vs G7 *P* = 0.5600 G5 vs G8 *P* = 0.0237 G5 vs G9 *P* = 0.2623 G6 vs G7 *P* = 0.0003 G6 vs G8 *P* < 0.0001 G6 vs G9 *P* < 0.0001 G7 vs G8 *P* = 0.5464 G7 vs G9 *P* = 0.9986 G8 vs G9 *P* = 0.8644	Ordinary one‐way ANOVA, Turkey's multiple comparison test
2	B	G2 vs G3 *P* = 0.8418 G2 vs G4 *P* = 0.0434 G2 vs G5 *P* > 0.9999 G2 vs G6 *P* = 0.7538 G2 vs G7 *P* = 3572 G2 vs G8 *P* < 0.0001 G2 vs G9 *P* = 0.0004 G3 vs G4 *P* = 0.4367 G3 vs G5 *P* = 0.8702 G3 vs G6 *P* > 0.9999 G3 vs G7 *P* = 0.0324 G3 vs G8 *P* < 0.0001 G3 vs G9 *P* < 0.0001	G4 vs G5 *P* = 0.0491 G4 vs G6 *P* = 0.5364 G4 vs G7 *P* = 0.0005 G4 vs G8 *P* < 0.0001 G4 vs G9 *P* < 0.0001 G5 vs G6 *P* = 0.7887 G5 vs G7 *P* = 0.3259 G5 vs G8 *P* < 0.0001 G5 vs G9 *P* = 0.0003 G6 vs G7 *P* = 0.0231 G6 vs G8 *P* < 0.0001 G6 vs G9 *P* < 0.0001 G7 vs G8 *P* < 0.0001 G7 vs G9 *P* = 0.0310 G8 vs G9 *P* = 0.0511	Ordinary one‐way ANOVA, Turkey's multiple comparison test
	D	G2 vs G3 *P* = 0.0766 G2 vs G4 *P* = 0.9127 G2 vs G5 *P* = 0.2621 G2 vs G6 *P* = 0.9990 G2 vs G7 *P* < 0.0001 G2 vs G8 *P* < 0.0001 G2 vs G9 *P* < 0.0001 G3 vs G4 *P* = 0.0074 G3 vs G5 *P* = 0.9945 G3 vs G6 *P* = 0.1995 G3 vs G7 *P* = 0.0013 G3 vs G8 *P* = 0.0002 G3 vs G9 *P* = 0.0023	G4 vs G5 *P* = 0.0304 G4 vs G6 *P* = 0.6404 G4 vs G7 *P* < 0.0001 G4 vs G8 *P* < 0.0001 G4 vs G9 *P* < 0.0001 G5 vs G6 *P* = 0.5428 G5 vs G7 *P* = 0.0003 G5 vs G8 *P* < 0.0001 G5 vs G9 *P* = 0.0006 G6 vs G7 *P* < 0.0001 G6 vs G8 *P* < 0.0001 G6 vs G9 *P* < 0.0001 G7 vs G8 *P* = 0.9698 G7 vs G9 *P* > 0.9999 G8 vs G9 *P* = 0.8987	Ordinary one‐way ANOVA, Turkey's multiple comparison test
3	B	G2 vs G3 *P* = 0.3762 G2 vs G4 *P* < 0.0001 G2 vs G5 *P* = 0.4787 G2 vs G6 *P* = 0.0034 G2 vs G7 *P* = 0.7715 G2 vs G8 *P* = 0.9482 G2 vs G9 *P* = 0. 9886 G3 vs G4 *P* = 0.0024 G3 vs G5 *P* > 0.9999 G3 vs G6 *P* = 0.2238 G3 vs G7 *P* = 0.9958 G3 vs G8 *P* = 0.0622 G3 vs G9 *P* = 1013	G4 vs G5 *P* = 0.0017 G4 vs G6 *P* = 0.2925 G4 vs G7 *P* = 0.0006 G4 vs G8 *P* < 0.0001 G4 vs G9 *P* < 0.0001 G5 vs G6 *P* = 0.1643 G5 vs G7 *P* = 0.9994 G5 vs G8 *P* = 0.0881 G5 vs G9 *P* = 0.1413 G6 vs G7 *P* = 0.0674 G6 vs G8 *P* = 0.0004 G6 vs G9 *P* = 0.0007 G7 vs G8 *P* = 0.2090 G7 vs G9 *P* = 0.3141 G8 vs G9 *P* > 0.9999	Ordinary one‐way ANOVA, Turkey's multiple comparison test
	C	G2 vs G3 *P* = 0.2128 G2 vs G4 *P* < 0.0001 G2 vs G5 *P* = 0.7482 G2 vs G6 *P* = 0.0004 G2 vs G7 *P* = 0.5080 G2 vs G8 *P* = 0.7121 G2 vs G9 *P* = 0.9192 G3 vs G4 *P* = 0.0043 G3 vs G5 *P* = 0.9600 G3 vs G6 *P* = 0.0639 G3 vs G7 *P* = 0.9978 G3 vs G8 *P* = 0.0101 G3 vs G9 *P* = 0.0244	G4 vs G5 *P* = 0.0006 G4 vs G6 *P* = 0.8418 G4 vs G7 *P* = 0.0013 G4 vs G8 *P* < 0.0001 G4 vs G9 *P* < 0.0001 G5 vs G6 *P* = 0.0086 G5 vs G7 *P* = 0.9999 G5 vs G8 *P* = 0.0741 G5 vs G9 *P* = 0.1636 G6 vs G7 *P* = 0.0193 G6 vs G8 *P* < 0.0001 G6 vs G9 *P* < 0.0001 G7 vs G8 *P* = 0.0343 G7 vs G9 *P* = 0.0797 G8 vs G9 *P* = 0.9997	Ordinary one‐way ANOVA, Turkey's multiple comparison test
3	E	G2 vs G3 *P* = 0.6901 G2 vs G4 *P* = 0.0016 G2 vs G5 *P* = 0.4511 G2 vs G6 *P* = 0.0003 G2 vs G7 *P* = 0.0005 G2 vs G8 *P* = 0.7692 G2 vs G9 *P* = 0.9893 G3 vs G4 *P* = 0.0428 G3 vs G5 *P* = 0.9999 G3 vs G6 *P* = 0.0071 G3 vs G7 *P* = 0.0123 G3 vs G8 *P* > 0.9999 G3 vs G9 *P* = 0.9843	G4 vs G5 *P* = 0.0910 G4 vs G6 *P* = 0.9783 G4 vs G7 *P* = 0.9973 G4 vs G8 *P* = 0.0327 G4 vs G9 *P* = 0.0079 G5 vs G6 *P* = 0.0158 G5 vs G7 *P* = 0.0271 G5 vs G8 *P* = 0.9990 G5 vs G9 *P* = 0.8914 G6 vs G7 *P* > 0.9999 G6 vs G8 *P* = 0.0054 G6 vs G9 *P* = 0.0013 G7 vs G8 *P* = 0.0093 G7 vs G9 *P* = 0.0022 G8 vs G9 *P* = 0.9944	Ordinary one‐way ANOVA, Turkey's multiple comparison test
	F	G2 vs G3 *P* > 0.9999 G2 vs G4 *P* = 0.0020 G2 vs G5 *P* = 0.8556 G2 vs G6 *P* < 0.0001 G2 vs G7 *P* = 0.5711 G2 vs G8 *P* > 0.9999 G2 vs G9 *P* = 0.9995 G3 vs G4 *P* = 0.0017 G3 vs G5 *P* = 0.8118 G3 vs G6 *P* < 0.0001 G3 vs G7 *P* = 0.5154 G3 vs G8 *P* > 0.9999 G3 vs G9 *P* = 0.9985	G4 vs G5 *P* = 0.0292 G4 vs G6 *P* = 0.4882 G4 vs G7 *P* = 0.0775 G4 vs G8 *P* = 0.0023 G4 vs G9 *P* = 0.0053 G5 vs G6 *P* = 0.0006 G5 vs G7 *P* = 0.9993 G5 vs G8 *P* = 0.8845 G5 vs G9 *P* = 0.9842 G6 vs G7 *P* = 0.0015 G6 vs G8 *P* < 0.0001 G6 vs G9 *P* = 0.0001 G7 vs G8 *P* = 0.6134 G7 vs G9 *P* = 0.8451 G8 vs G9 *P* = 0.9998	Ordinary one‐way ANOVA, Turkey's multiple comparison test
4	B	G2 vs G3 *P* < 0.0001 G2 vs G4 *P* < 0.0001 G2 vs G5 *P* > 0.9999 G2 vs G6 *P* < 0.0001 G2 vs G7 *P* < 0.0001 G2 vs G8 *P* = 0.0486 G2 vs G9 *P* = 0.0996 G3 vs G4 *P* = 0.9993 G3 vs G5 *P* < 0.0001 G3 vs G6 *P* = 0.9796 G3 vs G7 *P* > 0.9999 G3 vs G8 *P* < 0.0001 G3 vs G9 *P* < 0.0001	G4 vs G5 *P* < 0.0001 G4 vs G6 *P* = 0.9999 G4 vs G7 *P* = 0.9967 G4 vs G8 p < 0.0001 G4 vs G9 *P* < 0.0001 G5 vs G6 v0.0001 G5 vs G7 *P* < 0.0001 G5 vs G8 *P* = 0.0813 G5 vs G9 *P* = 0.1611 G6 vs G7 *P* = 0.9560 G6 vs G8 *P* < 0.0001 G6 vs G9 *P* < 0.0001 G7 vs G8 *P* < 0.0001 G7 vs G9 *P* < 0.0001 G8 vs G9 *P* = 0.9999	Ordinary one‐way ANOVA, Turkey's multiple comparison test
	C	G2 vs G3 *P* < 0.0001 G2 vs G4 *P* < 0.0001 G2 vs G5 *P* > 0.9999 G2 vs G6 *P* < 0.0001 G2 vs G7 *P* < 0.0001 G2 vs G8 *P* = 0. 1992 G2 vs G9 *P* = 0.3713 G3 vs G4 *P* = 0.5128 G3 vs G5 *P* < 0.0001 G3 vs G6 *P* = 0.4046 G3 vs G7 *P* > 0.9999 G3 vs G8 *P* < 0.0001 G3 vs G9 *P* < 0.0001	G4 vs G5 *P* < 0.0001 G4 vs G6 *P* > 0.9999 G4 vs G7 *P* = 0.4644 G4 vs G8 *P* < 0.0001 G4 vs G9 *P* < 0.0001 G5 vs G6 *P* < 0.0001 G5 vs G7 *P* < 0.0001 G5 vs G8 *P* = 0.1824 G5 vs G9 *P* = 0.3448 G6 vs G7 *P* = 0.3614 G6 vs G8 *P* < 0.0001 G6 vs G9 *P* < 0.0001 G7 vs G8 *P* < 0.0001 G7 vs G9 *P* < 0.0001 G8 vs G9 *P* = 0.9998	Ordinary one‐way ANOVA, Turkey's multiple comparison test
4	E	G2 vs G3 *P* = 0.0684 G2 vs G4 *P* = 0.0007 G2 vs G5 *P* = 0.9978 G2 vs G6 *P* = 0.0006 G2 vs G7 *P* = 0.0595 G2 vs G8 *P* = 0.0624 G2 vs G9 *P* = 0.9969 G3 vs G4 *P* = 0.2983 G3 vs G5 *P* = 0.0208 G3 vs G6 *P* = 0.2875 G3 vs G7 *P* > 0.9999 G3 vs G8 *P* = 0.0001 G3 vs G9 *P* = 0.0194	G4 vs G5 *P* = 0.0002 G4 vs G6 *P* > 0.9999 G4 vs G7 *P* = 0.3322 G4 vs G8 *P* < 0.0001 G4 vs G9 *P* = 0.0002 G5 vs G6 *P* = 0.0002 G5 vs G7 *P* = 0.0180 G5 vs G8 *P* = 0.1873 G5 vs G9 *P* > 0.9999 G6 vs G7 *P* = 0.3206 G6 vs G8 *P* < 0.0001 G6 vs G9 *P* = 0.0002 G7 vs G8 *P* = 0.0001 G7 vs G9 *P* = 0.0167 G8 vs G9 *P* = 0.1990	Ordinary one‐way ANOVA, Turkey's multiple comparison test
	F	G2 vs G3 *P* = 0.1671 G2 vs G4 *P* = 0.0007 G2 vs G5 *P* = 0.9997 G2 vs G6 *P* = 0.0007 G2 vs G7 *P* = 0.1148 G2 vs G8 *P* = 0.1662 G2 vs G9 *P* = 0.9986 G3 vs G4 *P* = 0.1428 G3 vs G5 *P* = 0.0747 G3 vs G6 *P* = 0.1412 G3 vs G7 *P* > 0.9999 G3 vs G8 *P* = 0.0009 G3 vs G9 *P* = 0.0595	G4 vs G5 *P* = 0.0003 G4 vs G6 *P* > 0.9999 G4 vs G7 *P* = 0.2055 G4 vs G8 *P* < 0.0001 G4 vs G9 *P* = 0.0002 G5 vs G6 *P* = 0.0003 G5 vs G7 *P* = 0.0497 G5 vs G8 *P* = 0.3373 G5 vs G9 *P* > 0.9999 G6 vs G7 *P* = 0.2033 G6 vs G8 *P* < 0.0001 G6 vs G9 *P* = 0.0002 G7 vs G8 *P* = 0.0006 G7 vs G9 *P* = 0.0394 G8 vs G9 *P* = 0.3985	Ordinary one‐way ANOVA, Turkey's multiple comparison test
5	B	G2 vs G3 *P* = 0.3021 G2 vs G4 *P* < 0.0001 G2 vs G5 *P* = 0.7210 G2 vs G6 *P* = 0.0006 G2 vs G7 *P* = 0.4331 G2 vs G8 *P* = 0.7928 G2 vs G9 *P* = 0.9609 G3 vs G4 *P* = 0.0073 G3 vs G5 *P* = 0.9929 G3 vs G6 *P* = 0.0612 G3 vs G7 *P* > 0.9999 G3 vs G8 *P* = 0.0212 G3 vs G9 *P* = 0.0519	G4 vs G5 *P* = 0.0017 G4 vs G6 *P* = 0.9455 G4 vs G7 *P* = 0.0043 G4 vs G8 *P* < 0.0001 G4 vs G9 *P* < 0.0001 G5 vs G6 *P* = 0.0143 G5 vs G7 *P* = 0.9995 G5 vs G8 *P* = 0.0888 G5 vs G9 *P* = 0.1983 G6 vs G7 *P* = 0.0371 G6 vs G8 *P* < 0.0001 G6 vs G9 *P* < 0.0001 G7 vs G8 *P* = 0.0353 G7 vs G9 *P* = 0.0846 G8 vs G9 *P* = 0.9997	Ordinary one‐way ANOVA, Turkey's multiple comparison test
	C	G2 vs G3 *P* = 0.1023 G2 vs G4 *P* = 0.0481 G2 vs G5 *P* > 0.9999 G2 vs G6 *P* = 0.1705 G2 vs G7 *P* = 0.9999 G2 vs G8 *P* = 0.0075 G2 vs G9 *P* = 0.0016 G3 vs G4 p = 0.9999 G3 vs G5 *P* = 0.0676 G3 vs G6 *P* > 0.9999 G3 vs G7 *P* = 0.2053 G3 vs G8 *P* < 0.0001 G3 vs G9 *P* < 0.0001	G4 vs G5 *P* = 0.0312 G4 vs G6 *P* = 0.9954 G4 vs G7 *P* = 0.1023 G4 vs G8 *P* < 0.0001 G4 vs G9 *P* < 0.0001 G5 vs G6 *P* = 0.1154 G5 vs G7 *P* = 0.9975 G5 vs G8 *P* = 0.0118 G5 vs G9 *P* = 0.0025 G6 vs G7 *P* = 0.3220 G6 vs G8 *P* < 0.0001 G6 vs G9 *P* < 0.0001 G7 vs G8 *P* = 0.0034 G7 vs G9 *P* = 0.0007 G8 vs G9 *P* = 0.9905	Ordinary one‐way ANOVA, Turkey's multiple comparison test
5	E	G2 vs G3 *P* = 0.9992 G2 vs G4 *P* = 0.0186 G2 vs G5 *P* = 0.9768 G2 vs G6 *P* < 0.0001 G2 vs G7 *P* > 0.9999 G2 vs G8 *P* = 0.9999 G2 vs G9 *P* > 0.9999 G3 vs G4 *P* = 0.0066 G3 vs G5 *P* = 0.8103 G3 vs G6 *P* < 0.0001 G3 vs G7 *P* = 0.9871 G3 vs G8 *P* > 0.9999 G3 vs G9 *P* = 0.9841	G4 vs G5 *P* = 0.1078 G4 vs G6 *P* = 0.0339 G4 vs G7 *P* = 0.0339 G4 vs G8 *P* = 0.0084 G4 vs G9 *P* = 0.0360 G5 vs G6 *P* = 0.0001 G5 vs G7 *P* = 0.9979 G5 vs G8 *P* = 0.8671 G5 vs G9 *P* = 0.9984 G6 vs G7 *P* < 0.0001 G6 vs G8 *P* < 0.0001 G6 vs G9 *P* < 0.0001 G7 vs G8 *P* = 0.9949 G7 vs G9 *P* > 0.9999 G8 vs G9 *P* = 0.9935	Ordinary one‐way ANOVA, Turkey's multiple comparison test
	F	G2 vs G3 *P* = 0.0152 G2 vs G4 *P* = 0.9958 G2 vs G5 *P* = 0.9991 G2 vs G6 *P* = 0.0425 G2 vs G7 *P* = 0.0074 G2 vs G8 *P* = 0.1046 G2 vs G9 *P* > 0.9999 G3 vs G4 *P* = 0.0577 G3 vs G5 *P* = 0.0434 G3 vs G6 *P* < 0.0001 G3 vs G7 *P* > 0.9999 G3 vs G8 *P* = 0.9631 G3 vs G9 *P* = 0.0108	G4 vs G5 *P* > 0.9999 G4 vs G6 *P* = 0.0110 G4 vs G7 *P* = 0.0286 G4 vs G8 *P* = 0.3226 G4 vs G9 *P* = 0.9845 G5 vs G6 *P* = 0.0149 G5 vs G7 *P* = 0.0213 G5 vs G8 *P* = 0.2578 G5 vs G9 *P* = 0.9950 G6 vs G7 *P* < 0.0001 G6 vs G8 *P* = 0.0001 G6 vs G9 *P* = 0.0589 G7 vs G8 *P* = 0.8457 G7 vs G9 *P* = 0.0052 G8 vs G9 *P* = 0.0765	Ordinary one‐way ANOVA, Turkey's multiple comparison test

**Table 4:** Statistics for Supplementary figure 1.

Figure	Subfigure	*P*‐value		Stats Test
1	D	G1 vs G2 *P* < 0.0001 G1 vs G3 *P* < 0.0001 G1 vs G4 *P* < 0.0001 G1 vs G5 *P* < 0.0001 G1 vs G6 *P* < 0.0001 G1 vs G7 *P* < 0.0001 G1 vs G8 *P* < 0.0001 G1 vs G9 *P* < 0.0001 G2 vs G3 *P* = 0.8641 G2 vs G4 *P* = 0.0004 G2 vs G5 *P* = 0.0263 G2 vs G6 *P* = 0.7816 G2 vs G7 *P* = 0.8831 G2 vs G8 *P* = 0.0775 G2 vs G9 *P* = 0.2464 G3 vs G4 *P* = 0.0085 G3 vs G5 *P* = 0.3507 G3 vs G6 *P* > 0.9999	G3 vs G7 *P* = 0.1671 G3 vs G8 *P* = 0.0041 G3 vs G9 *P* = 0.0163 G4 vs G5 *P* = 0.5616 G4 vs G6 *P* = 0.0122 G4 vs G7 *P* < 0.0001 G4 vs G8 *P* < 0.0001 G4 vs G9 *P* < 0.0001 G5 vs G6 *P* = 0.4421 G5 vs G7 *P* = 0.0015 G5 vs G8 *P* < 0.0001 G5 vs G9 *P* = 0.0001 G6 vs G7 *P* = 0.1233 G6 vs G8 *P* = 0.0029 G6 vs G9 *P* = 0.0114 G7 vs G8 *P* = 0.6376 G7 vs G9 *P* = 0.9432 G8 vs G9 *P* = 0.9988	Ordinary one‐way ANOVA, Turkey's multiple comparison test
	E	G1 vs G2 *P* < 0.0001 G1 vs G3 *P* = 0.0054 G1 vs G4 *P* < 0.0001 G1 vs G5 *P* < 0.0001 G1 vs G6 *P* < 0.0001 G1 vs G7 *P* < 0.0001 G1 vs G8 *P* < 0.0001 G1 vs G9 *P* < 0.0001 G2 vs G3 *P* = 0.0263 G2 vs G4 *P* > 0.9999 G2 vs G5 *P* = 0.9929 G2 vs G6 *P* = 0.0586 G2 vs G7 *P* = 0.0044 G2 vs G8 *P* < 0.0001 G2 vs G9 *P* < 0.0001 G3 vs G4 *P* = 0.0404 G3 vs G5 *P* = 0.0045 G3 vs G6 *P* < 0.0001	G3 vs G7 *P* < 0.0001 G3 vs G8 *P* < 0.0001 G3 vs G9 *P* < 0.0001 G4 vs G5 *P* = 0.9718 G4 vs G6 *P* = 0.0385 G4 vs G7 *P* = 0.0028 G4 vs G8 *P* < 0.0001 G4 vs G9 *P* < 0.0001 G5 vs G6 *P* = 0.2656 G5 vs G7 *P* = 0.0257 G5 vs G8 *P* < 0.0001 G5 vs G9 *P* < 0.0001 G6 vs G7 *P* = 0.9274 G6 vs G8 *P* < 0.0001 G6 vs G9 *P* < 0.0001 G7 vs G8 *P* < 0.0001 G7 vs G9 *P* < 0.0001 G8 vs G9 *P* = 0.0037	Ordinary one‐way ANOVA, Turkey's multiple comparison test

*Note*: All comparisons and respective *P*‐values reported, along with statistical test performed.

## AUTHOR CONTRIBUTIONS

Juliana Restrepo Munera co‐designed the study, executed all experiments, analyzed the data, and co‐wrote the manuscript. S. Rameeza Allie Co‐designed the study, helped in data interpretation, and the writing of the manuscript.

## CONFLICT OF INTEREST STATEMENT

No conflict of interest to disclose.

## Supporting information


Supplementary figure 1.

Supplementary figure 2.

## Data Availability

The data that support the findings of this study are openly available in ScholarSphere at https://scholarsphere.psu.edu.
