# Supplementary figures and images for: IL‐1β and IL‐6 synergistically drive murine TFH cell differentiation and maintenance in vitro
Source: Immunol Cell Biol. 2026 Mar 31;104(5):457–72. doi: 10.1111/imcb.70104 (PMC13155040; doi:10.1111/imcb.70104)

**A.**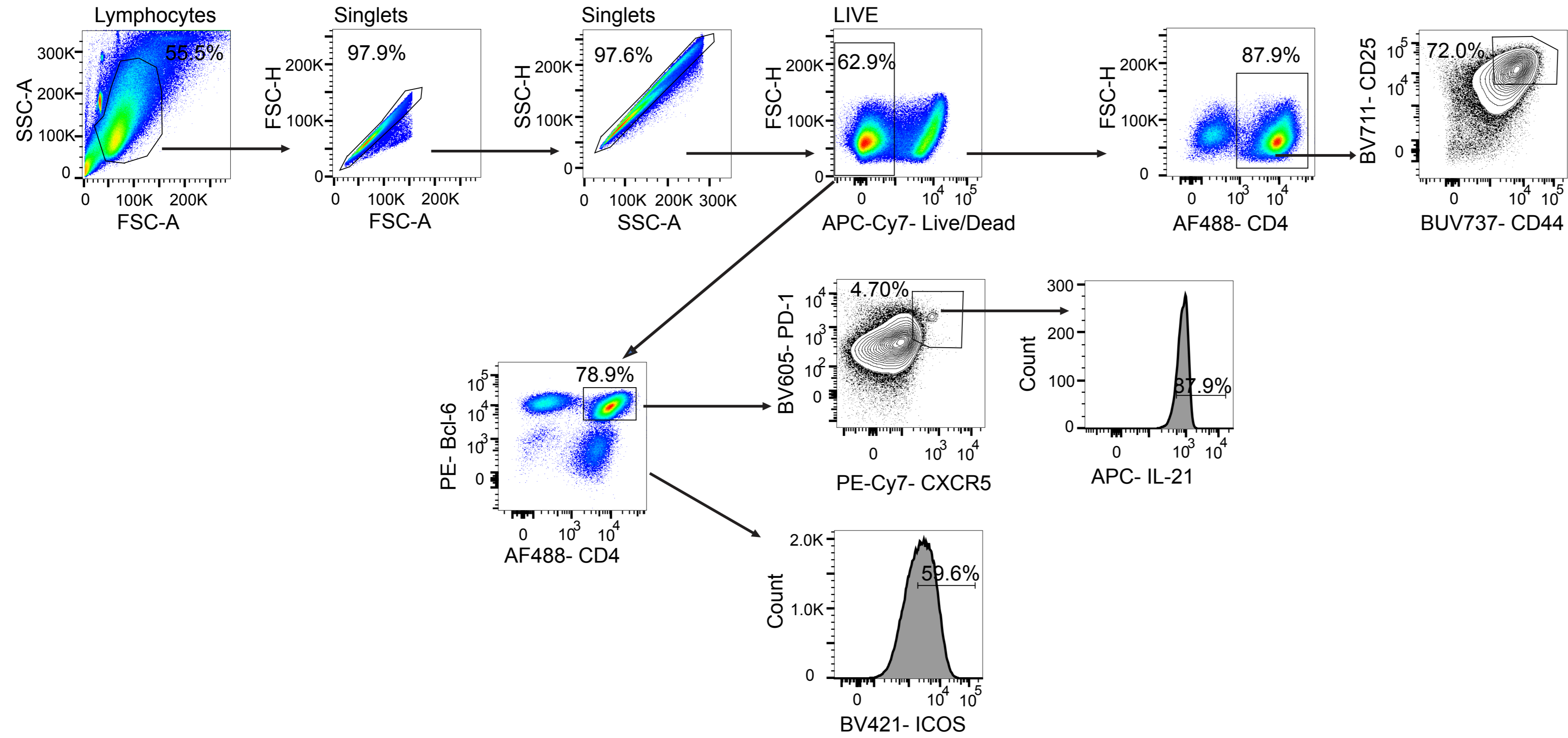**B.**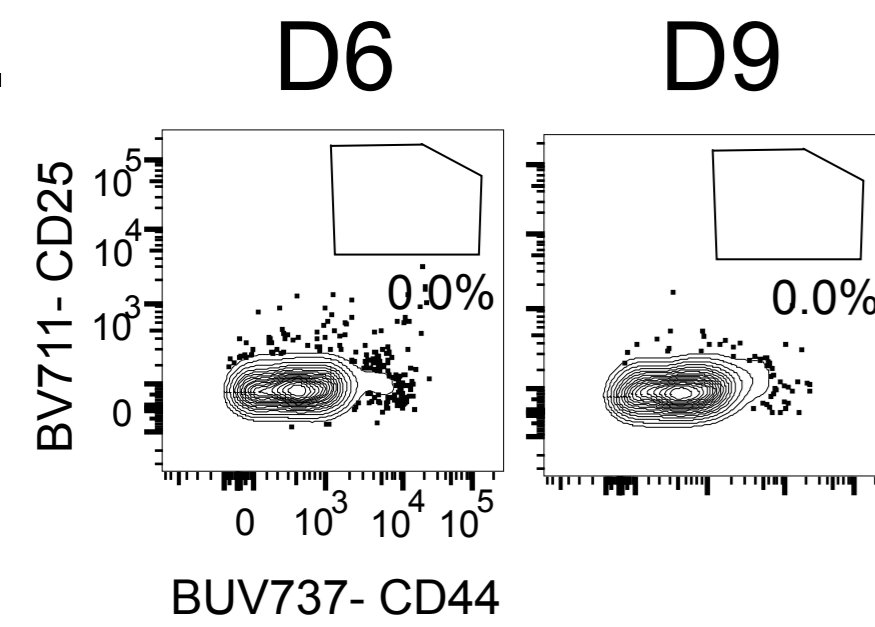**C.**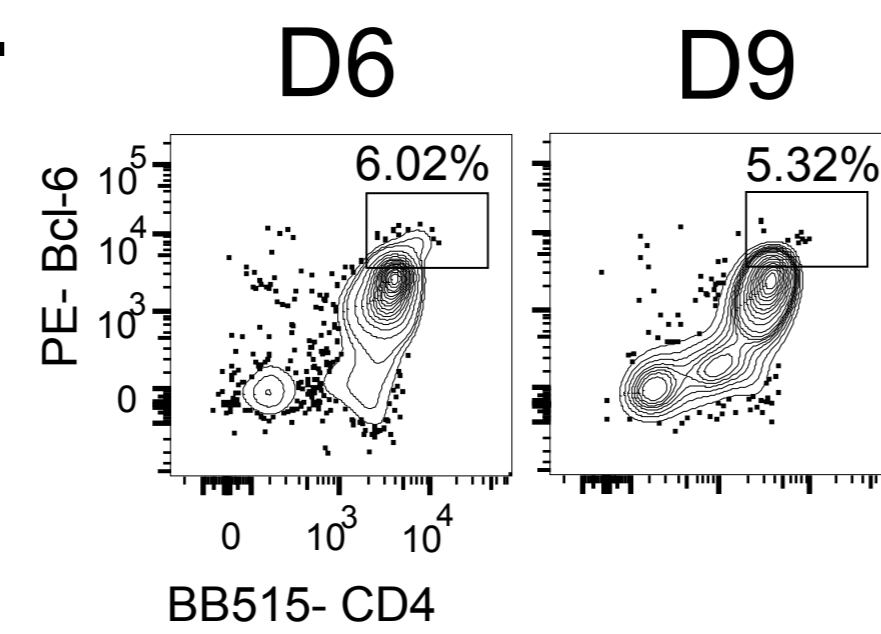**D.**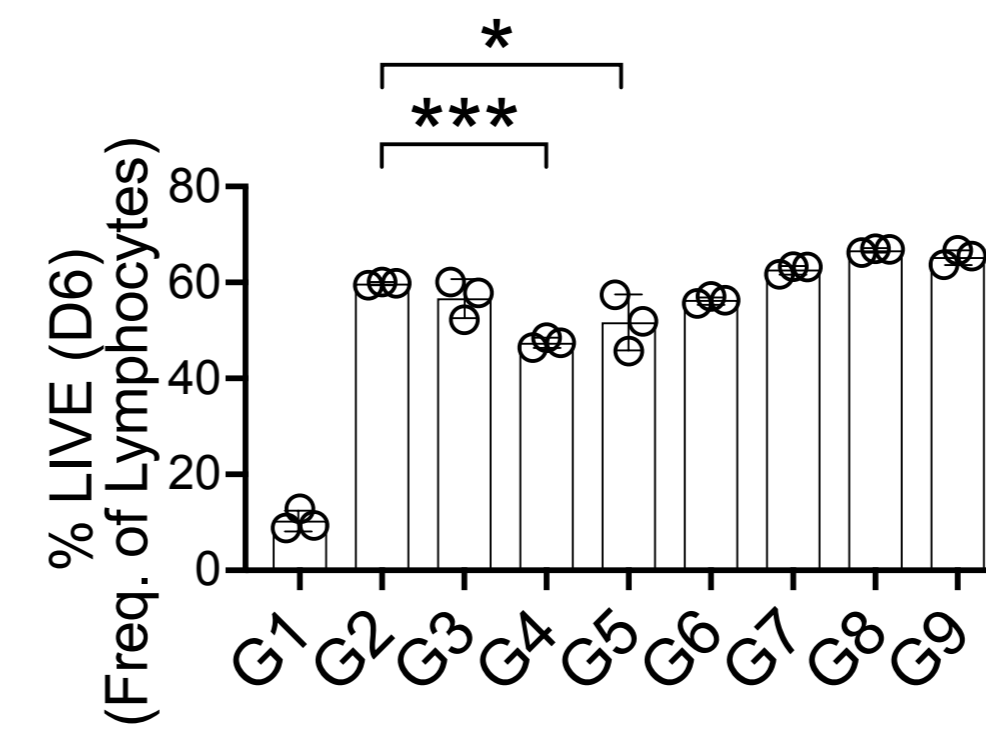**E.**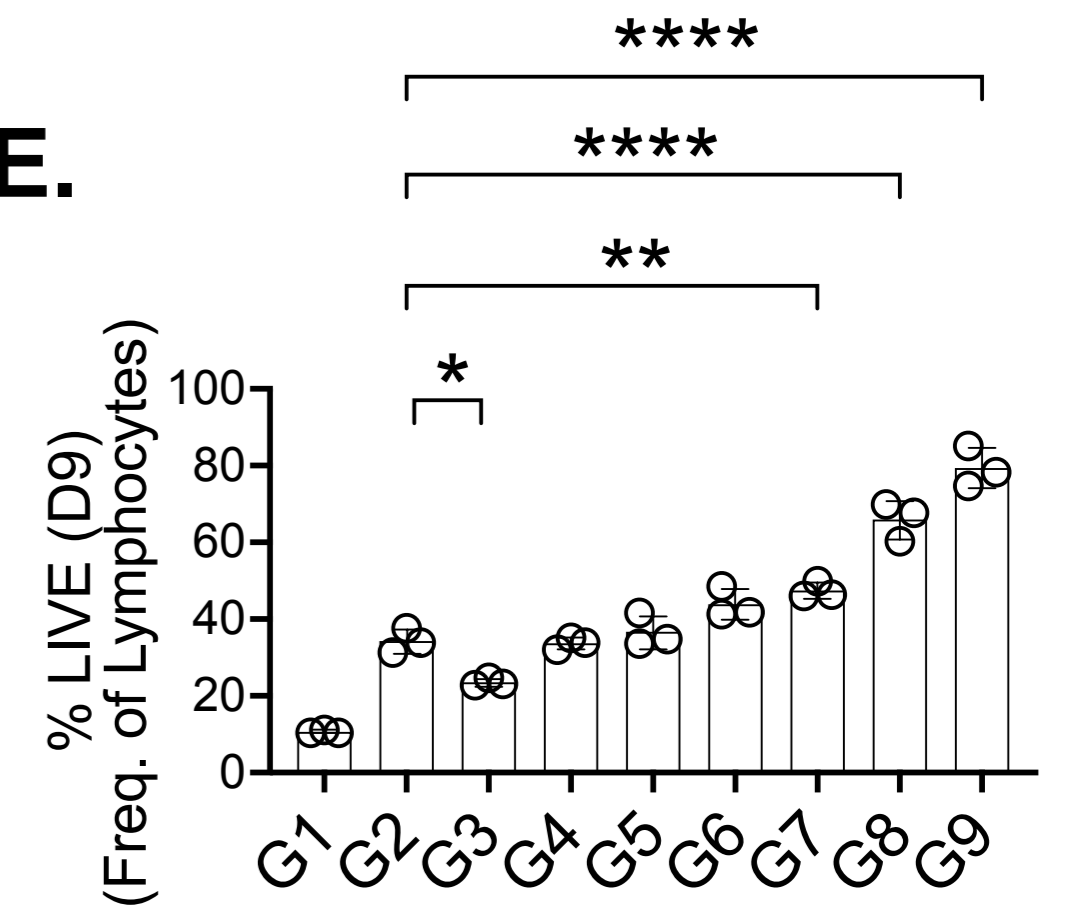

Supplement: Supplementary file 1 — Supplementary figure 1. [file IMCB-104-457-s002.pdf]
