# Supplementary material for: IL‐1β and IL‐6 synergistically drive murine TFH cell differentiation and maintenance in vitro
Source: Immunol Cell Biol. 2026 Mar 31;104(5):457–72. doi: 10.1111/imcb.70104 (PMC13155040; doi:10.1111/imcb.70104)

A.

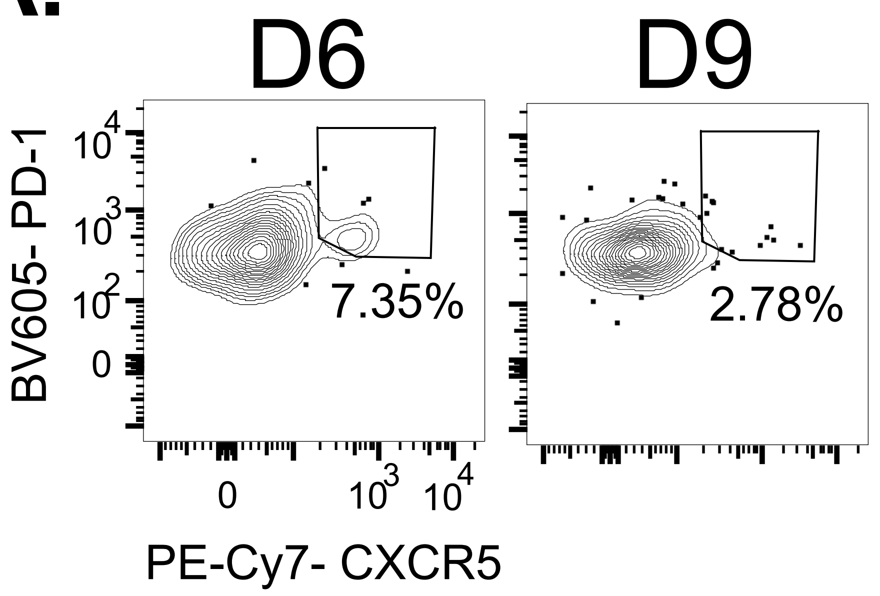

B.

$$\%\ \text{Lymphocytes} = \frac{\text{Singlets 1 X Singlets 2 X LIVE X Bcl-6+CD4+ X CXCR5+PD-1+}}{(100^{5})} \text{ X } 100$$

$$\%\ \text{Lymphocytes} = \frac{97.9 \text{ X } 97.6 \text{ X } 62.9 \text{ X } 78.9 \text{ X } 4.70}{(100^{5})} \text{ X } 100 = 2.23\%$$

C.

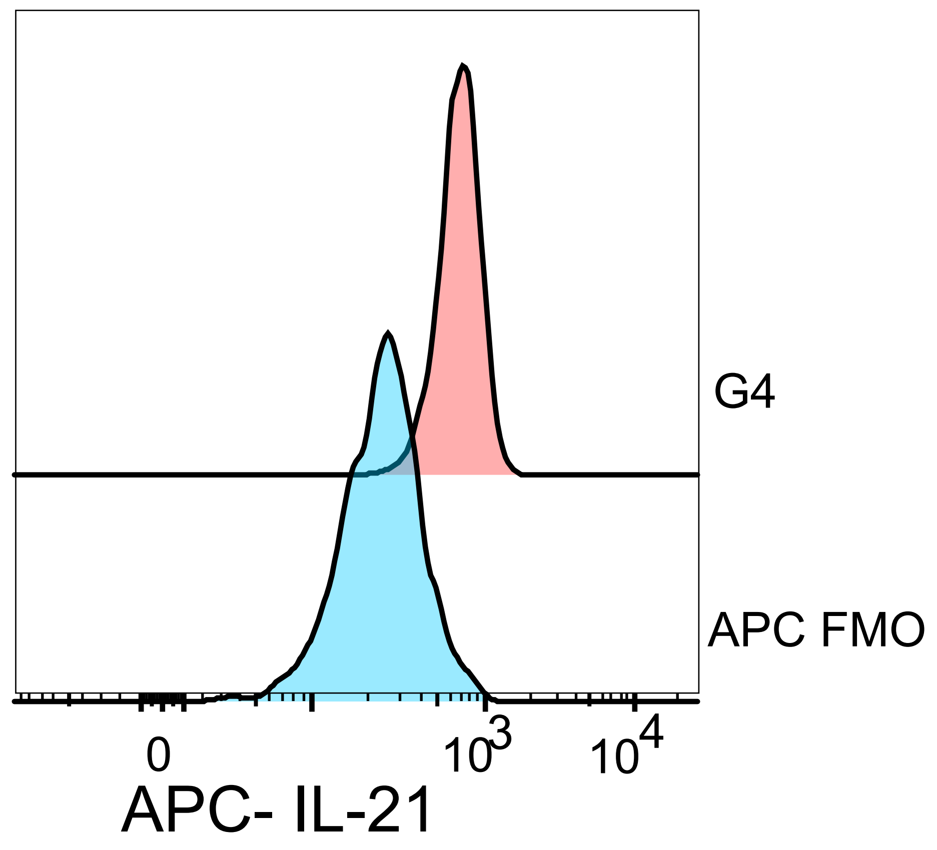

Supplement: Supplementary file 2 — Supplementary figure 2. [file IMCB-104-457-s001.pdf]
